# Impact of temporal resolution on perfusion metrics, therapy decision, and radiation dose reduction in brain CT perfusion in patients with suspected stroke

**DOI:** 10.1007/s00234-024-03335-w

**Published:** 2024-03-18

**Authors:** Alexander Rau, Marco Reisert, Thomas Stein, Katharina Mueller-Peltzer, Stephan Rau, Fabian Bamberg, Christian A. Taschner, Horst Urbach, Elias Kellner

**Affiliations:** 1 Department of Neurology and Clinical Neuroscience, Medical Center – University of Freiburg, Faculty of Medicine, University of Freiburg, Freiburg, Germany; 2 Department of Diagnostic and Interventional Radiology, Medical Center – University of Freiburg, Faculty of Medicine, University of Freiburg, Freiburg, Germany; 3 Department of Medical Physics, Medical Center – University of Freiburg, Faculty of Medicine, University of Freiburg, Freiburg, Germany; 4 Department of Stereotactic and Functional Neurosurgery, Medical Center – University of Freiburg, Faculty of Medicine, University of Freiburg, Freiburg, Germany

**Keywords:** Perfusion, Stroke, Radiation dosage, Computed tomography

## Abstract

**Purpose:**

CT perfusion of the brain is a powerful tool in stroke imaging, though the radiation dose is rather high. Several strategies for dose reduction have been proposed, including increasing the intervals between the dynamic scans. We determined the impact of temporal resolution on perfusion metrics, therapy decision, and radiation dose reduction in brain CT perfusion from a large dataset of patients with suspected stroke.

**Methods:**

We retrospectively included 3555 perfusion scans from our clinical routine dataset. All cases were processed using the perfusion software VEOcore with a standard sampling of 1.5 s, as well as simulated reduced temporal resolution of 3.0, 4.5, and 6.0 s by leaving out respective time points. The resulting perfusion maps and calculated volumes of infarct core and mismatch were compared quantitatively. Finally, hypothetical decisions for mechanical thrombectomy following the DEFUSE-3 criteria were compared.

**Results:**

The agreement between calculated volumes for core (ICC = 0.99, 0.99, and 0.98) and hypoperfusion (ICC = 0.99, 0.99, and 0.97) was excellent for all temporal sampling schemes. Of the 1226 cases with vascular occlusion, 14 (1%) for 3.0 s sampling, 23 (2%) for 4.5 s sampling, and 63 (5%) for 6.0 s sampling would have been treated differently if the DEFUSE-3 criteria had been applied. Reduction of temporal resolution to 3.0 s, 4.5 s, and 6.0 s reduced the radiation dose by a factor of 2, 3, or 4.

**Conclusion:**

Reducing the temporal sampling of brain perfusion CT has only a minor impact on image quality and treatment decision, but significantly reduces the radiation dose to that of standard non-contrast CT.

**Supplementary Information:**

The online version contains supplementary material available at 10.1007/s00234-024-03335-w.

## Introduction

In most centers, indication for therapy of an acute ischemic stroke is based on multimodal CT imaging protocols that comprise non-contrast CT, CT-angiography, and CT perfusion (CTP) imaging. CTP is a powerful tool in stroke imaging with proven high accuracy for the detection of ischemic lesions and the differentiation of infarct core from potentially salvageable brain tissue, the so-called mismatch volume. CTP was successfully used as the selection criterion for endovascular therapy in important trials, showing benefit for patients with limited-size-predicted infarct core or substantial salvageable brain tissue [1, 2]. Therefore, current stroke guidelines require CTP in patients with extended and unclear time windows [3].

Despite the paramount benefit in the selection of stroke therapy and the high diagnostic value, the radiation dose of current default protocols is rather high [4–6]. The radiation exposure is determined by various parameters, including tube current, tube voltage, slice coverage, temporal acquisition window, and temporal sampling interval. Consequently, multiple strategies have been proposed for dose reduction [4], in particular by increasing the temporal sampling intervals [7–12]. In retrospective modeling in small patient series, the principle feasibility of a lower temporal sampling of the perfusion bolus was shown: Wiesmann et al. observed that the diagnostic value of the calculated perfusion maps at a temporal resolution of 3.0 s is equivalent to the original data set with 1.0 s [8]. This is also reflected in the results of Abels and colleagues, who observed no difference between 1.0 s and 2.0 s temporal resolution [11]. In a recent study, Ma et al. demonstrated that a resolution up to 5.1 s still yields reliable results in terms of quantification of the core-penumbra mismatch [13].

Consequently, reduced temporal resolution constitutes a promising approach for radiation dose reduction. To evaluate the feasibility of this concept for clinical routine and assess potential pitfalls, we investigated the impact of increasing the intervals from 1.5 to 6.0 s based on 3555 perfusion scans from clinical routine. Here, we hypothesized that radiation dose reduction via increased temporal sampling intervals does not substantially hamper CTP metrics and therapy decisions.

## Methods

### Participants

For this retrospective study, we included patients that received neuroimaging including CTP due to a suspected stroke between 2014 and 2021. All scan data were exported and anonymized to a local instance of the in-house imaging platform NORA (www.nora-imaging.org). The platform is tailored for storage, management, batch processing, visualization, and rating of medical images in a web-based environment, with a specific focus on handling large datasets. Patient records and image data were investigated to determine if a large or medium vessel occlusion was present. The study was approved by the Institutional Review Board (Ethics Committee – University of Freiburg; EK 20/1047) and carried out in accordance with the Declaration of Helsinki and its later amendments. Due to the retrospective nature of this study, the need for written informed consent was waived.

### CT imaging

CT scans were performed on a 128-detector row (Somatom Definition Flash; Siemens) or a 64-detector row CT scanner (Somatom Definition 64 AS; Siemens). CTP series were acquired in the axial scan mode with the following protocols for the Somatom Definition Flash: 80 kV, 180 mAs, collimation = 16 × 1.2 mm, no gantry tilt, tube rotation time = 0.3 s, spiral mode, *z*-coverage = 100 mm, slice thickness = 5 mm, 27 frames every 1.5 s = 42 s scan time (effective temporal resolution in the middle of the slab = 1.5 s, in the periphery = 2.6 s) with 5 s delay after intravenous (16–18 G) injection of 40 mL of Imeron 400 (iopamidol; Bracco) + 30 mL of sodium chloride (NaCl) at a flow rate of 6 mL/s.

The parameters for Somatom Definition 64 AS were the following: 80 kV, 180 mAs, collimation = 32 × 1.2 mm, no gantry tilt, tube rotation time = 0.3 s, spiral mode, *z*-coverage = 90 mm, slice thickness = 5 mm, 30 frames every 1.5 s = 45 s scan time (effective temporal resolution in the middle of the slab = 1.5 s, in the periphery = 2.6 s) with 5 s delay after intravenous (16–18 G) injection of 40 mL of Imeron 400 + 30 mL of NaCl at a flow rate of 6 mL/s.

For all protocols, images reconstructed to 5-mm slices were used for perfusion processing.

### Perfusion processing

Perfusion scans were analyzed using the software package VEOcore (VEObrain GmbH, www.veobrain.com). The software provides fully automated perfusion processing including motion correction, denoising, deconvolution using Tikhonov regularization, and automated quality control. The outputs of the software are perfusion maps of cerebral blood flow (CBF), cerebral blood volume (CBV), and time to maximum of the residue function (*T*max) where CBF and CBV are normalized to the full contralateral hemisphere on a patient-individual basis. Additionally, the software provides automated segmentation and volumetry of hypoperfusion and core using the commonly established thresholds of *T*max > 6 s for hypoperfusion and CBF < 30% for the infarct core [1, 14, 15]. Further, the mismatch volume (i.e., penumbra) defined as hypoperfusion volume minus core volume, and the mismatch ratio defined as the ratio of hypoperfusion and core volume were calculated. All cases were processed with the reference sampling of 1.5 s and simulated reduced temporal resolution of 3.0 s, 4.5 s, and 6.0 s by leaving out respective time points. Good agreement of VEOcore with other perfusion software has been demonstrated [16, 17].

#### Quality control

Acquisition of CT perfusion data can be hampered, e.g., due to incomplete contrast administration, truncated boli, and heavy patient motion [18]. This can significantly impact the interpretability of perfusion results, ranging from limited assessment to complete uninterpretability. It can be expected that the issues become more severe with reduced temporal sampling. To investigate this, we performed a quality control based on both visual and automated measures. First, results for the reference sampling with 1.5 s were rated visually, and cases with obviously non-interpretable results due to fatal errors, such as completely truncated boli, failed contrast injection or extreme patient movement, were excluded. For the remaining cases, we used the automated quality control system provided by the VEOcore software (VEObrain GmbH, www.veobrain.com). This system analyzes the mean contrast bolus curve using several metrics and creates a notification if values are suspicious. In this study, we employed the following thresholds: temporal bolus peak position later than 90% of the acquisition window (to identify truncated boli), bolus peak height smaller than 8 HU (to identify cases with low contrast-to-noise ratio), and motion index (defined as the correlation of each dynamic scan with the first scan) smaller than 0.7 (to identify cases with strong motion). Cases without artifacts detected visually or automatically were assigned to a subset of high scan quality, and cases with artifact notifications were assigned to a subset of potentially impaired quality.

### Calculation of radiation doses

All dose calculations (CTDI_vol_ and DLP) were performed using a commercially available dose management and reporting platform (DoseM, INFINITT Europe GmbH, Frankfurt, Germany). Doses for the 3.0 s, 4.5 s, and 6.0 s were obtained by division with the proportion of timepoints required for the respective sampling scheme.

### Statistical analyses

To assess the interpretability of increased temporal sampling, the original sampling with 1.5 s served as a reference and was compared with simulated 3.0 s, 4.5 s, and 6.0 s. The correlations between the calculated volumes of infarct core, hypoperfusion, mismatch, and the mismatch ratio were plotted as correlation- and bland–altman plots and quantified in terms of intraclass-correlation-coefficient (ICC), and median and standard deviation of the difference of ischemic volumes (ml) between the reference and reduced sampling schemes. The median was chosen to account for a potentially non-Gaussian distribution. For the calculation of the mismatch ratio, small core volumes might result in singular values [13]. Hence, only cases with core volume > 3 mL were considered for analyzing the mismatch ratio to obtain interpretable results [19].

As a qualitative measure of comparison, the cases were sorted according to the differences in core- and hypoperfusion volume with respect to the 1.5 s reference protocol. The 50 topmost cases with very good agreement (i.e., small differences) and low agreement (i.e., great differences) were visually inspected in order to identify reasons for outliers.

Finally, as an additional quantitative measure, in the subgroup with vessel occlusions, hypothetical decisions for mechanical thrombectomy following the DEFUSE-3 criteria [1] were compared. The DEFUSE-3 criteria state that mechanical thrombectomy is appropriate if the CBF core is smaller than 70 mL, the mismatch volume is greater than 15 mL, and the mismatch ratio (hypoperfusion/core) is larger than 1.8. Hypothetical treatment decisions were generated for the reference sampling with 1.5 s and for all other samplings based on these thresholds. The precision of every measurement is inherently limited due to noise and other factors. Consequently, for values in close proximity to the thresholds for treatment decisions, the decision criteria become indistinct. It becomes challenging to discern, for instance, whether a volume of 72 mL truly differs from 69 mL. To mitigate this ambiguity, we estimated a precision of 5 mL for the volumes and 0.2 for the mismatch ratio and applied a corresponding error margin when applying the thresholds. In other words, cases with core volumes between 65 and 75 mL, mismatch volumes between 10 and 20 mL, and mismatch ratios between 1.6 and 2.0 were excluded in the comparison with the confusion matrix. This systematic approach enhances the reliability of our analysis by minimizing the impact of measurement uncertainties and ensuring a more robust evaluation of the data. Upon applying the thresholds for sampling intervals of 3.0 s, 4.5 s, and 6.0 s, we adjusted the boundaries by these amounts to account for the measurement precision. The results were evaluated using a confusion matrix, which provides a summary of the counts for true positives, false positives, true negatives, and false negatives in relation to the 1.5 s reference. All statistical analyses were performed using MATLAB (The MathWorks, Natick, USA).

### Data availability

Data is available upon reasonable request and approval by the local ethics committee.

## Results

### Participants

In this retrospective monocentric study, we report on 3555 cases with CTP of the brain from our clinical routine dataset (*n* = 2249 scanned with the Somatom Flash and *n* = 1306 scanned with the Somatom Definition AS). Vessel occlusion was diagnosed in 1226 patients. Based on visual quality control of the reference images with 1.5 s sampling, 274 cases were excluded from further analysis due to fatal technical issues (i.e., very strong motion artifacts, very low raw scan data quality, or incomplete contrast injection, an example is given in the Supplementary Fig. 1). From the remaining 3281 cases (mean age of 73 ± 15 years; 1754 (53.5%) female), the automated quality control identified 3015 cases with high quality and 266 with impaired scan quality. Mean volumes were 30.4 ± 45.4 mL for infarct core, 76.1 ± 86.5 mL for hypoperfusion, and 61.3 ± 72.4 mL for mismatch volume. The mean mismatch ratio was 8.0 ± 12.4.

### Impact of temporal resolution on perfusion metrics

**Fig. 1:**
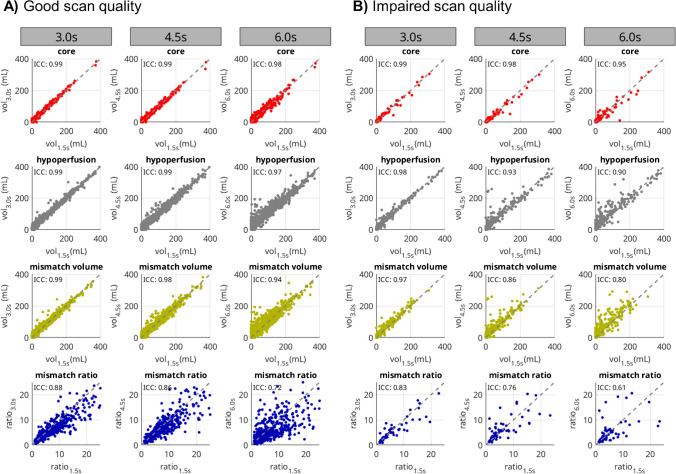
Correlations between the reference sampling of 1.5 s and simulated 3.0 s, 4.5 s, and 6.0 s samplings for core-, hypoperfusion-, and mismatch-volumes as well as the mismatch ratio. The left three columns **A** show the subset of cases with good scan data quality, right three columns **B** with impaired quality

Correlation plots are given in Fig. 1, and the corresponding Bland–Altman plots are provided in Supplementary Fig. 2. For the subset with high scan quality, there is excellent agreement in core- and mismatch volumes for all sampling schemes (0.99, 0.99, and 0.98 for core and 0.99, 0.99, and 0.97 for hypoperfusion, respectively). In the subset with impaired scan quality, we noted excellent agreement, too (0.99, 0.98, and 0.95 for core and 0.99, 0.93, and 0.90 for hypoperfusion, respectively). ICC and standard deviation of the volume differences were smaller in the subset with high scan quality compared to impaired scan quality, indicating greater variability and more outliers for the latter (see Fig. 1).

**Fig. 2:**
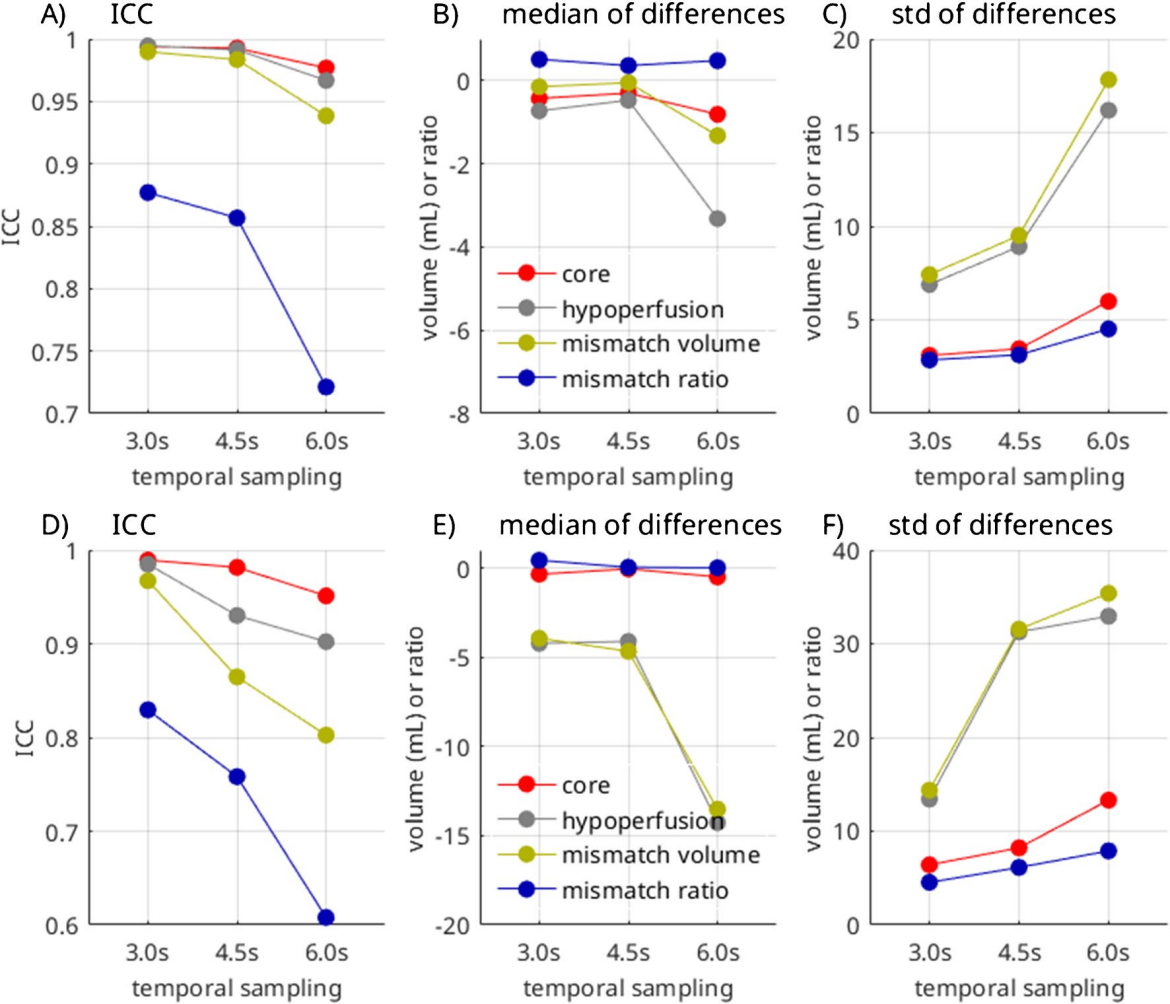
Intra-class correlation coefficient (ICC), median of the differences, and standard deviation of the differences for the different temporal samplings for the group with good quality **A**–**C** and impaired quality **D**–**F**. The decreasing ICC and increasing deviation reflect that higher temporal sampling comes at the cost of lower measurement precision

This is also reflected in Fig. 2, which graphically illustrates the evolution of the correlation metrics with increasing temporal resolution, indicating an exponential increase in ICC and standard deviation for volumes of core, hypoperfusion, and mismatch, as well as mismatch ratio. The median values show a more stable pattern, indicating that there is no systematic bias when increasing the sampling intervals.

**Fig. 3:**
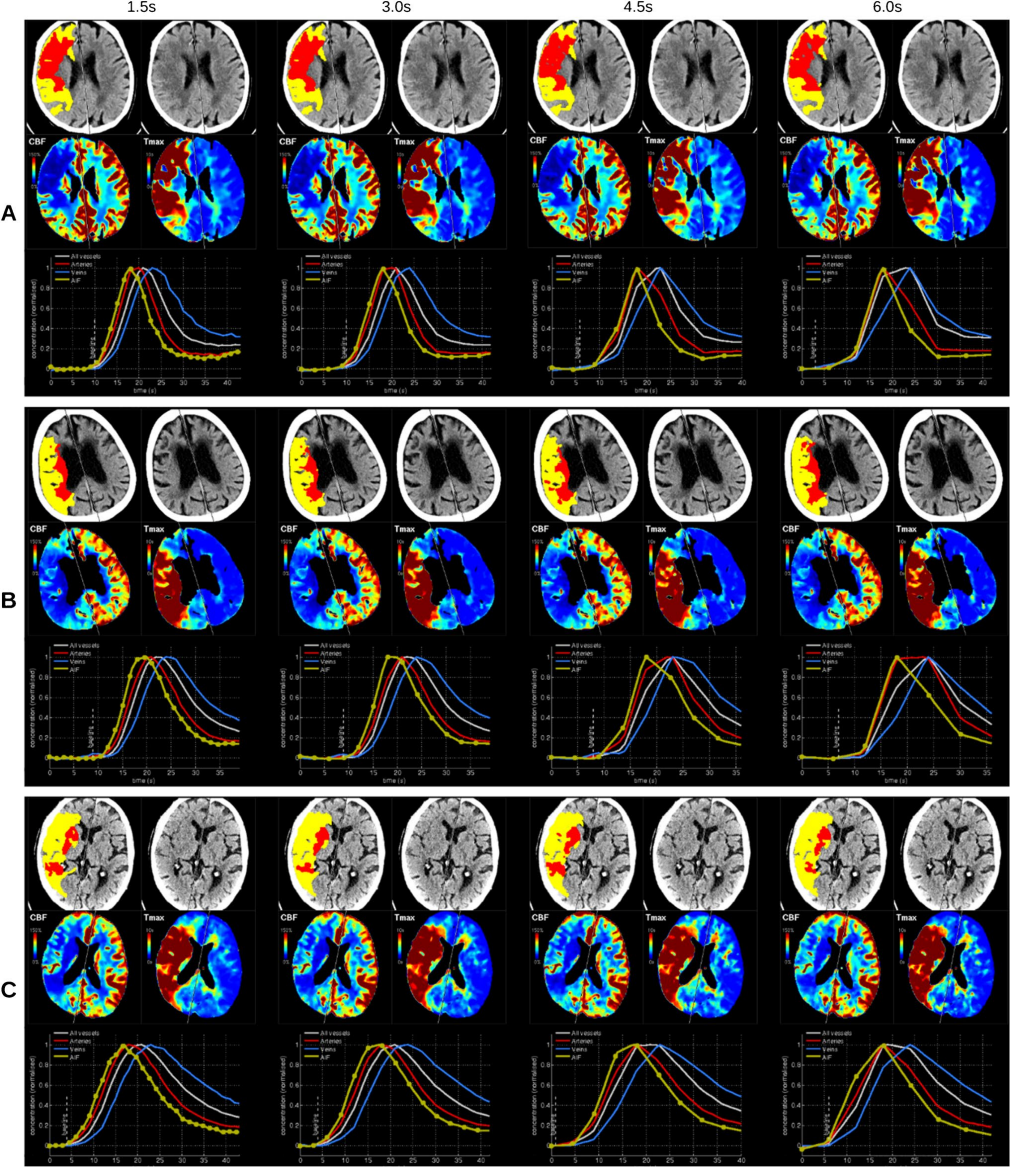
Representative perfusion maps of cerebral blood flow (CBF) and time-to-maximum (*T*max) as well as time-concentration-curves in cases with good scan quality for all sampling intervals. In most cases, there is almost no difference, both in the visual perception of the perfusion maps as well as in the segmentations of core (red) and mismatch volume (yellow) (**A** and **B**). In some cases, the *T*max maps present with slightly enhanced noise at the highest sampling interval, however, without an impact on the diagnostic usability (**C**)

**Fig. 4:**
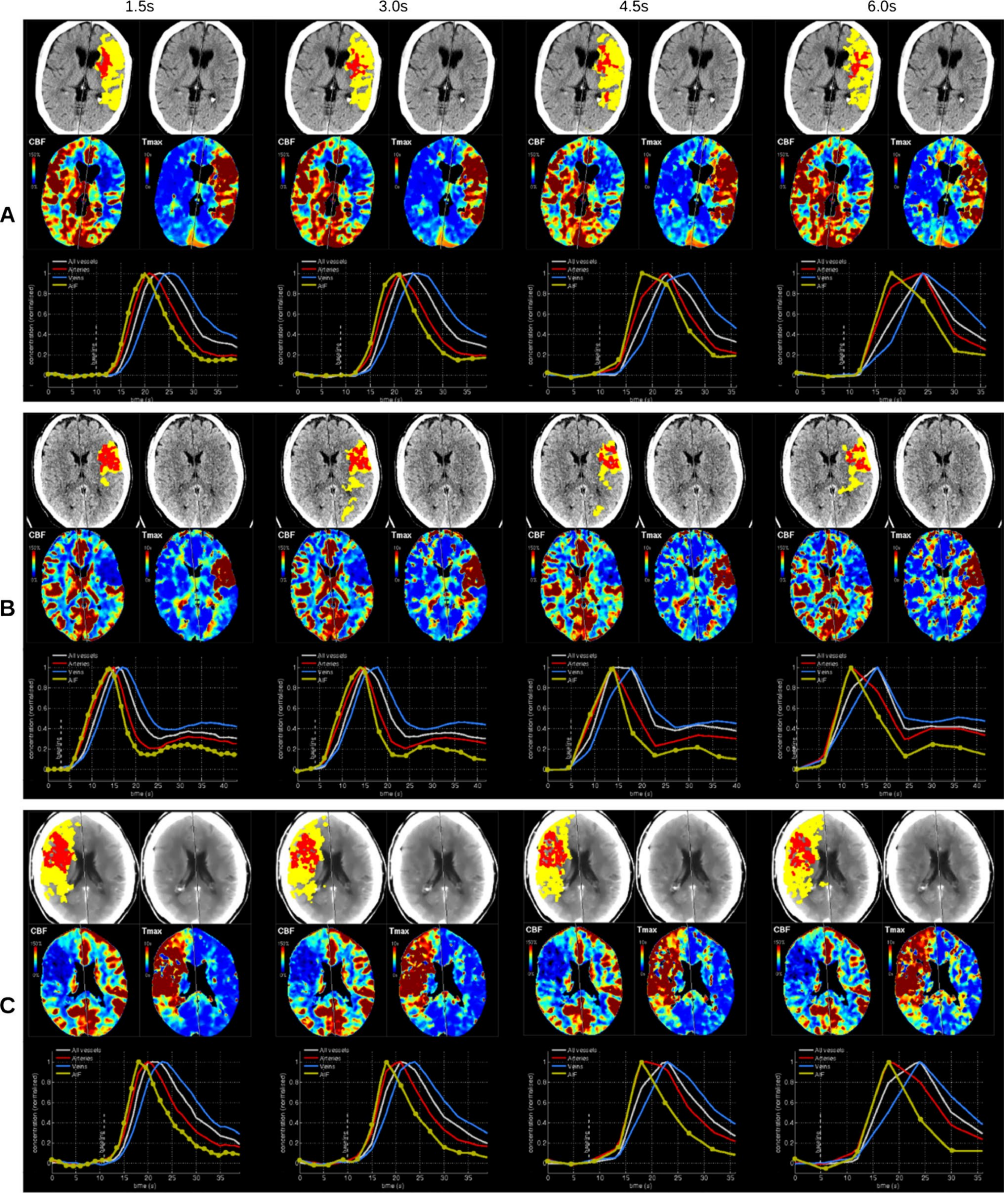
Representative perfusion maps of cerebral blood flow (CBF) and time-to-maximum (*T*max) as well as time-concentration-curves in cases with impaired scan quality. Issues in the scans (low contrast-to-noise, late bolus, motion) can lead to increased noise and slight differences in the segmentations at higher sampling rates. However, the diagnostic quality of the results is usually still sufficient in these cases

Representative examples for both the groups with good and impaired scan quality are provided in Figs. 3 and 4. In the group with high scan quality, we did not observe a visually noticeable difference even for a sampling interval up to 6.0 s (Fig. 3). Visual inspection of cases with greater differences (i.e., outliers in the correlation plots) suggests that greater differences were more common for a narrow shape of the contrast bolus (Supplemental Fig. 3).

Visual inspection of examples of the group with impaired scan quality indicated increased noise in the perfusion maps at higher sampling intervals (Fig. 4). Quantitatively, this resulted in greater differences in the infarct core and mismatch volume segmentations (Fig. 1).

### Treatment decisions

Outcomes for the hypothetical treatment decision with respect to the DEFUSE-3 criteria are summarized in the confusion matrix in Table 1. Of the 1226 cases of vascular occlusion, 14 (1%) for 3.0 s sampling, 23 (2%) for 4.5 s sampling, and 63 (5%) for 6.0 s sampling would have been treated differently.

**Table 1:** Hypothetical treatment decisions based on the DEFUSE-3 criteria

				**1.5 s**		**3.0 s**		**4.5 s**		**6.0 s**	
				** + **	**-**	** + **	**-**	** + **	**-**	** + **	**-**
**1.5 s (*n*)**	** + **	**TP**	**FN**	869	0	863	6	861	8	847	22
	**-**	**FP**	**TN**	0	357	8	349	15	342	41	316
**1.5 s (%)**	** + **	**TP**	**FN**	100%	0%	99%	1%	99%	1%	97%	3%
	**-**	**FP**	**TN**	0%	100%	2%	98%	4%	96%	11%	89%

Plus and minus signs represent decisions for or against mechanical thrombectomy, respectively. Relying on 1.5 s as a reference, the confusion matrices show true positives (TP), false positives (FP), true negatives (TN), and false negatives (TN, FN) for all samplings in absolute units (to rows) and percent (bottom row)

### Radiation dose

A comparison of the radiation doses of NCCT, CTA, and CTP with 1.5 s and simulated reduced temporal sampling is provided in Table 2. The reference sampling of 1.5 s corresponds to a radiation dose of CTDI_vol_: 210 mGy and 4.6 mSv effective dose for a 10 cm scan range for the Somatom Flash and a CTDI_vol_: 259 mGy and 5.11 mSv effective dose for a 9 cm scan range for the Somatom Definition AS. With increasing sampling intervals, the dose can be reduced by a factor of around 2, 3, and 4, respectively.

**Table 2:** Parameters and radiation doses of a multimodal CT stroke protocol

		**NCCT**	**CTA**	**CTP**			
	Subsampling factor	–	–	1	2	3	4
	Sampling interval	–	–	1.5 s	3.0 s	4.5 s	6.0 s
Somatom flash	Number of timepoints	–	–	27	14	9	7
	Slice coverage (mm)	160	320	100	100	100	100
	CTDIvol [mGy]	45	6.5	210	109	70	54
	DLP [mGy*cm]	724	207	2103	1091	701	545
	Effective Dose (mSv)	1.59	1.13	4.61	2.51	1.61	1.25
Somatom definition AS	Number of timepoints	–	–	30	15	10	8
	Slice coverage (mm)	160	320	90	90	90	90
	CTDIvol [mGy]	45	7.7	259	129	86	69
	DLP [mGy*cm]	727	247	2331	1165	777	621
	Effective dose (mSv)	1.59	1.35	5.11	2.68	1.79	1.43

Doses for computed tomography perfusion (CTP) with simulation of increased temporal sampling were calculated by adjusting the reference protocol with 1.5 s to the respective number of timepoints. *NCCT* non-contrast computed tomography, *CTA* computed tomography angiography, *CTDIvol* computed tomography dose volume index, *DLP* dose length product

## Discussion

The concept of reducing the radiation dose of CTP by increased sampling intervals is not new. It is obvious that a tradeoff has to be found between sampling rate and radiation exposure. Consequently, the definition of the lowest feasible sampling rate is an ongoing debate. There has been a prevailing notion that a temporal sampling below 2 s is required [10, 12, 13]. Some studies reported miscalculation of perfusion values and underestimation of ischemic volume when increasing the temporal sampling beyond this point [7, 10]. However, other studies challenged this notion and demonstrated that intervals up to 5.1 s still yield robust results [8, 13]. In the present study, we investigated sampling intervals up to 6.0 s in a large dataset from a clinical routine. Our results indicate that increasing the temporal sampling from 1.5 up to 6.0 s seems feasible but naturally comes at the cost of decreased quality of the perfusion maps and reliability of calculated infarct volumes as discussed below. This resulted in a simulated reduction of the radiation dose of up to 75% for the sampling with 6.0 s and corresponds to a radiation dose as low as that of non-contrast CT of the brain. In contrast, in current multimodal examination protocols, CTP constitutes a considerable position in terms of radiation exposure [20–22]. However, the diagnostic value of imaging protocol benefits significantly from CTP, especially beyond the 4.5-h time window and without large vessel occlusions [1, 2, 23, 24].

Whereas previous studies on the concept of increased temporal sampling in CTP relied on rather small datasets [7–9, 11, 12], we examined a large dataset from a clinical routine which included a wide range of scan qualities. Here, we found that impaired data quality (for example due to motion, late contrast arrival, or low contrast to noise ratios) resulted in stronger artifacts when reducing the temporal resolution. This observation aligns logically, as one would anticipate greater distinctions in situations in which the starting scan quality is already compromised.

In terms of the variations in ischemic volumes across various sampling intervals, it is important to highlight that the differences we observed were within the range of comparing distinct CTP processing solutions among each other [25–31].

In the simulated decision-making process according to DEFUSE-3 criteria, we noted a higher proportion of hypothetically different treatment strategies with increased temporal sampling. However, it should be noted that imprecise decisions can occur when strictly adhering to DEFUSE-3 criteria, and the level of inconsistency was relatively low and within previously reported ranges [13]. Nevertheless, in a prospective clinical application of increased sampling intervals, particular caution in the interpretation of the results should be exercised so that possible thrombectomy decisions are not influenced to the disadvantage of the patient.

The data we included was from CT scanners of a single vendor and all were acquired in spiral (i.e. helical) mode. Two potential issues should be considered in this context: First, for the 1.5 s and 4.5 s protocol, the nominal sampling rate is only reached for the central slices, whereas the outer slices are sampled at a non-uniform, effectively lower rate. For the 3.0 and 6.0 s protocols, all slices are sampled at a constant, uniform rate since data is always acquired during the same movement direction in this case. However, in practical observations, we did not identify a systematic difference in this respect.

The second point to consider is that our results might not directly be applicable to data acquired with scanners of other vendors and acquisition modes such as jog mode [32, 33]. On the other hand, comparable results have been reported by others using scanners from different vendors [8, 13], supporting the generalizability of the results.

It should further be noted that for all sampling schemes, the timeseries started with the first datapoint but did not always end on the last data point available (27 and 30, respectively) since the latter were not a common multiple for all subsampling factors (1, 2, 3, and 4). This might have contributed to an additional variability in the results. Moreover, the total scan durations of 42 s and 45 s used in this study are rather short compared to a study suggesting scanning between 60 and 70 s [34]. However, this does not change the main message of the study: Cases with bolus truncations [5, 35] were excluded, and the relative dose reduction of up to 75% would be the same for longer acquisition windows.

For this study, our primary focus rested on analyzing the sampling interval while keeping the remaining parameters constant. The combination of reduced temporal resolution with other established dose reduction techniques such as reduced tube voltage or current or improved image denoising in post-processing using artificial intelligence has to be attributed to future research [36–38]. Such a synergistic approach may result in a tremendous improvement in patient safety while expanding the application of CTP. A holistic balancing of all parameters also opens the possibility of further increasing the stability and accuracy of perfusion results also for cases with impaired scan quality. For example, the known issue of bolus truncation [5, 35] (see Supplemental Fig. 1) can directly be solved by allocating some of the saved dose toward extending the acquisition window.

## Conclusion

Reducing the temporal sampling of brain perfusion CT has only a minor impact on image quality and treatment decision but significantly reduces the radiation dose to that of standard non-contrast CT.

## Supplementary information

### Supplementary Information

Below is the link to the electronic supplementary material.

Supplementary file1 (DOCX 5662 KB)
