# Supplementary material for: Impact of temporal resolution on perfusion metrics, therapy decision, and radiation dose reduction in brain CT perfusion in patients with suspected stroke
Source: Neuroradiology. 2024 Mar 18;66(5):749–59. doi: 10.1007/s00234-024-03335-w (PMC11031466; doi:10.1007/s00234-024-03335-w)
Supplement: Supplementary file 1 — Supplementary file1 (DOCX 5662 KB) [file 234_2024_3335_MOESM1_ESM.docx]

**Supplementary Material**

**
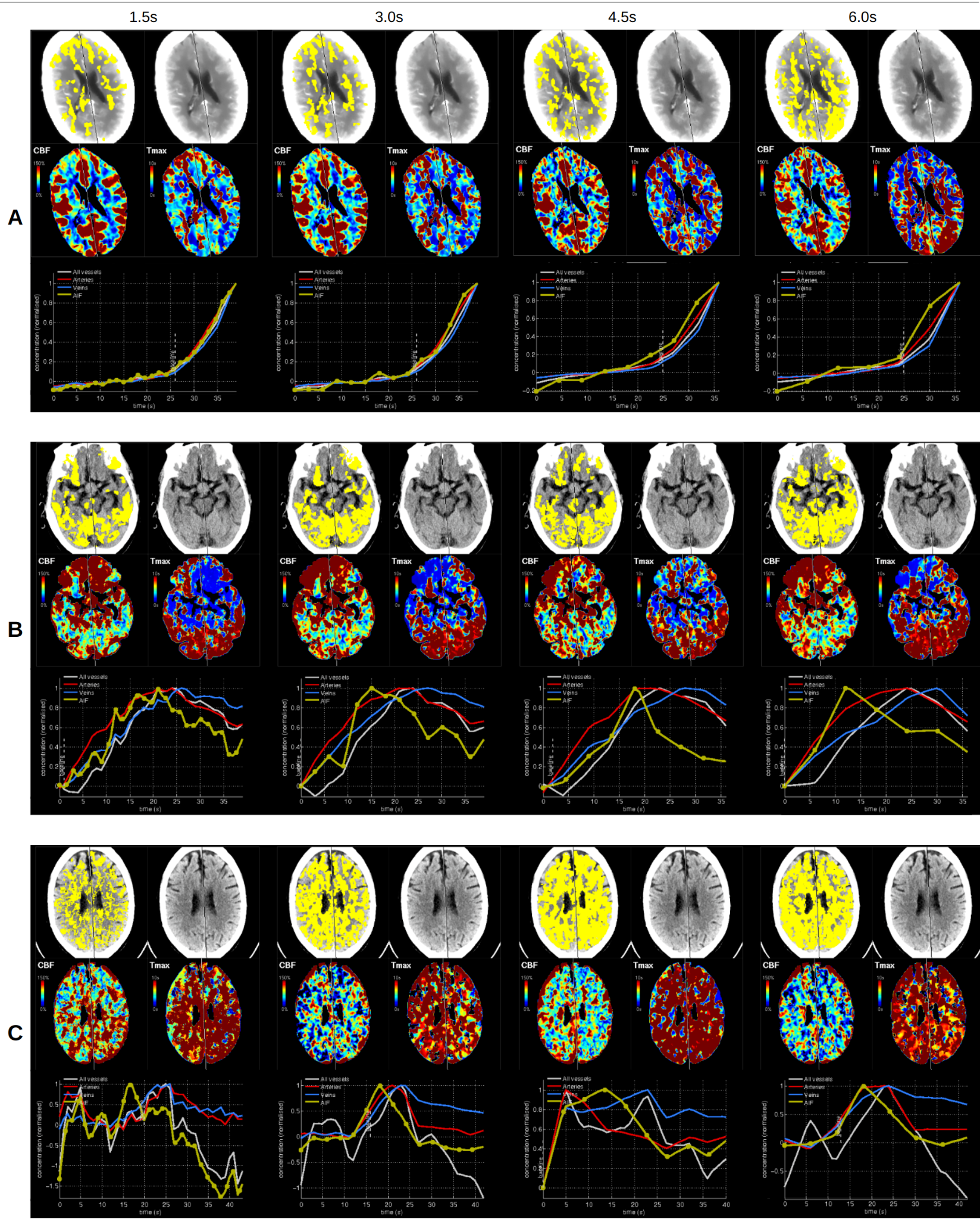
**

**Supplemental Figure 1** Exemplary perfusion maps of cerebral blood flow (CBF) and time-to-maximum (Tmax) as well as time-concentration-curves in cases of fatal quality, for example due to very late bolus arrival or failed contrast injection. Such cases were excluded from the analysis.

**A)** Good scan quality  **B)** Impaired scan quality


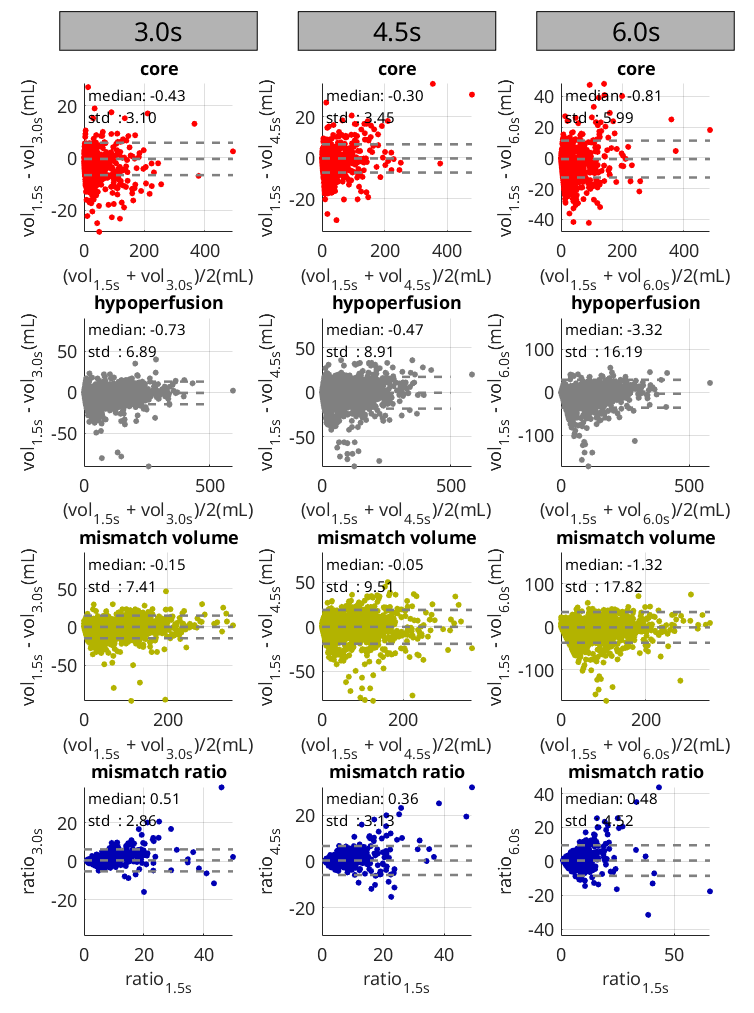

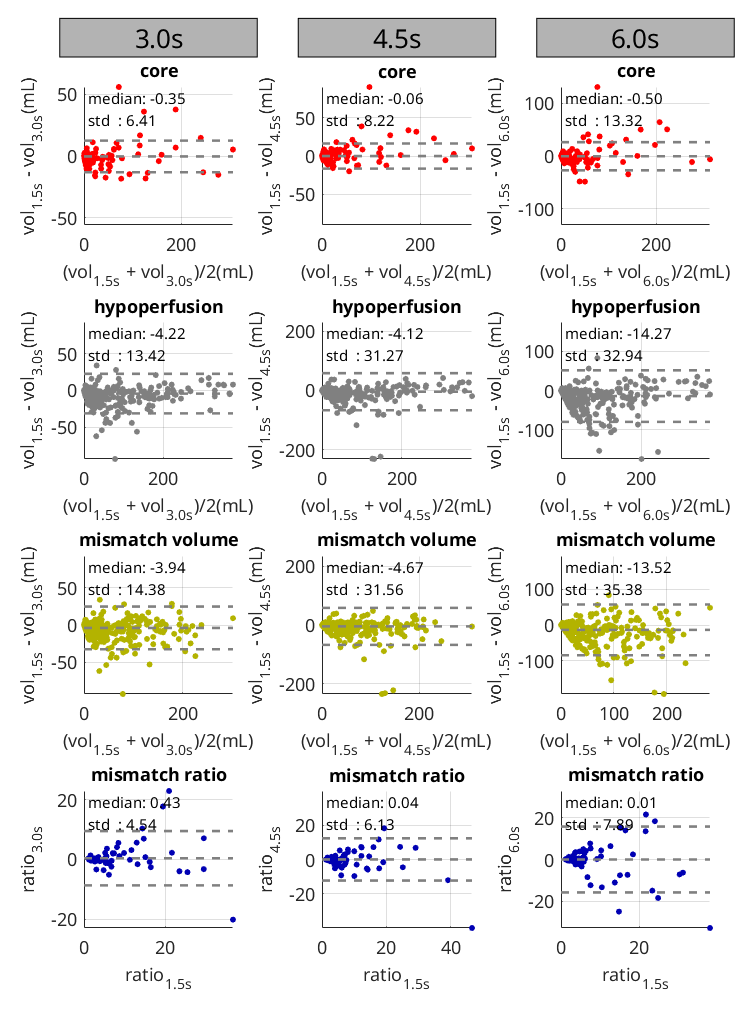


**Supplemental Figure 2** Bland-Altman plots comparing the reference sampling of 1.5s and simulated 3.0s, 4.5s, and 6.0s samplings for core-, hypoperfusion- and mismatch-volumes as well as the mismatch ratio. Left three columns (A) show the subset of cases with good scan data quality, right three columns (B) with impaired quality.

**
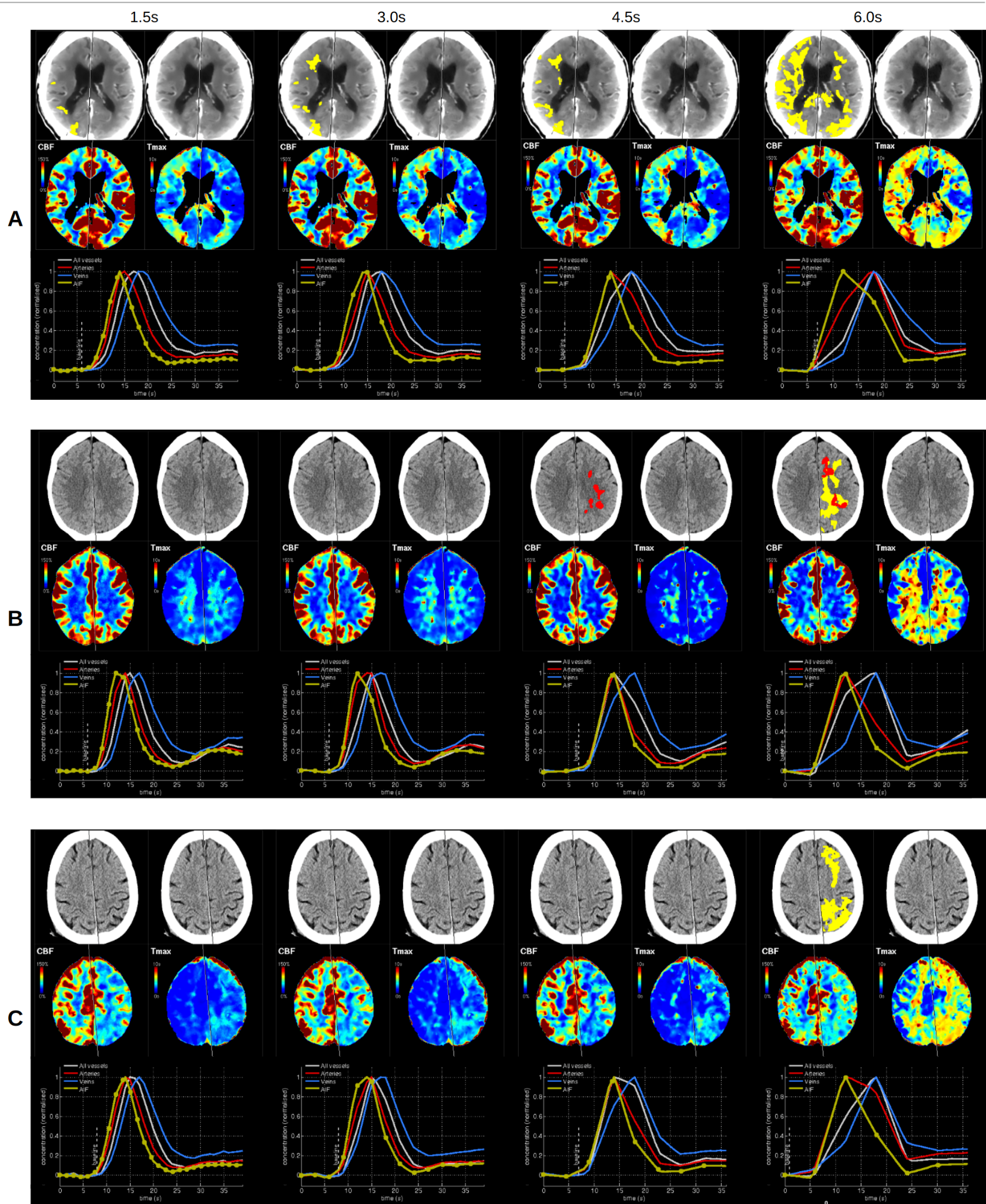
Supplemental Figure 3** Representative perfusion maps of cerebral blood flow (CBF) and time-to-maximum (Tmax) as well as time-concentration-curves in cases with good scan quality, but nevertheless noticeable differences at the highest sampling interval with 6.0s, especially in the Tmax maps. This occurred most often in cases with narrow contrast boli, where the sampling limit is reached first.
